# Genetic knockout of *NTRK2* by CRISPR/Cas9 decreases neurogenesis and favors glial progenitors during differentiation of neural progenitor stem cells

**DOI:** 10.3389/fncel.2023.1289966

**Published:** 2023-12-14

**Authors:** Audrey Roussel-Gervais, Stéphanie Sgroi, Yves Cambet, Sylvain Lemeille, Tamara Seredenina, Karl-Heinz Krause, Vincent Jaquet

**Affiliations:** ^1^Department of Pathology and Immunology, Faculty of Medicine, University of Geneva, Geneva, Switzerland; ^2^READS Unit, Faculty of Medicine, University of Geneva, Geneva, Switzerland

**Keywords:** neural differentiation, neural progenitor cells, tropomyosin receptor kinase B, brain-derived neurotrophic factor, CRISPR/Cas9

## Abstract

The tropomyosin receptor kinase B (TrkB) is encoded by the *NTRK2* gene. It belongs to the family of transmembrane tyrosine kinases, which have key roles in the development and maintenance of the nervous system. Brain-derived neurotrophic factor (BDNF) and the neurotrophins NT3 and NT4/5 have high affinity for TrkB. Dysregulation of TrkB is associated to a large spectrum of diseases including neurodegeneration, psychiatric diseases and some cancers. The function of TrkB and its role in neural development have mainly been decrypted using transgenic mouse models, pharmacological modulators and human neuronal cell lines overexpressing *NTRK2*. In this study, we identified high expression and robust activity of TrkB in ReNcell VM, an immortalized human neural progenitor stem cell line and generated *NTRK2*-deficient (*NTRK2*^–/–^) ReNcell VM using the CRISPR/Cas9 gene editing technology. Global transcriptomic analysis revealed major changes in expression of specific genes responsible for neurogenesis, neuronal development and glial differentiation. In particular, key neurogenic transcription factors were massively down-regulated in *NTRK2*^–/–^ cells, while early glial progenitor markers were enriched in *NTRK2*^–/–^ cells compared to *NTRK2*^+/+^. This indicates a previously undescribed inhibitory role of TrkB on glial differentiation in addition to its well-described pro-neurogenesis role. Altogether, we have generated for the first time a human neural cell line with a loss-of-function mutation of *NTRK2*, which represents a reproducible and readily available cell culture system to study the role of TrkB during human neural differentiation, analyze the role of TrkB isoforms as well as validate TrkB antibodies and pharmacological agents targeting the TrkB pathway.

## 1 Introduction

The human *NTRK2* gene encodes the Tropomyosin receptor kinase B (TrkB), a protein that plays a crucial role in neuronal development, function, and survival. TrkB is a transmembrane tyrosine kinase receptor with high affinity for brain-derived neurotrophic factor (BDNF), a neurotrophin that promotes the growth, survival, and differentiation of neurons in the central and peripheral nervous systems. TrkB comprises an extracellular ligand-binding domain, a transmembrane domain and an intracellular kinase domain (Li et al., 2022). However alternative splicing of the *NTRK2* gene can give rise to several splice variants, including the TrkB-T1 isoform that lacks the tyrosine kinase domain. TrkB isoforms have different functions and cellular specificities (Tessarollo and Yanpallewar, 2022). Upon BDNF binding, the full-length TrkB receptor undergoes dimerization and autophosphorylation on the intracellular kinase domain, leading to the recruitment of intracellular signaling molecules that transmit cellular growth and survival signals (Reichardt, 2006) and influence neuronal survival, differentiation, synaptic transmission, and plasticity, mechanisms that are essential for the proper development of the nervous system and the maintenance of neuronal health throughout life.

Mutations in the human *NTRK2* gene leads to various symptoms, including neurodevelopmental delay, epilepsy and autism (Yeo et al., 2004; Hamdan et al., 2017). Numerous reports indicate that dysregulation of TrkB function is critical in neurological disorders, such as depression, schizophrenia, neurodevelopmental disorders such as autism spectrum disorders and neurogenic tumors (Li et al., 2022). Accordingly, modulation of TrkB activity has high therapeutic potential for a wide range of diseases (Li et al., 2023). TrkB has recently gained new attention with the discovery that the antidepressant effect of psychedelic drugs is linked to positive allosteric modulation of TrkB (Casarotto et al., 2021). *NTRK2* is expressed in neural epithelium-derived tissues and has the highest expression in neurons, astrocytes and oligodendrocyte precursors (Sjöstedt et al., 2020); Human Protein Atlas proteinatlas.org. Targeted disruption of the mouse *Ntrk2* gene leads to severe deficiency of both central and peripheral nervous systems and neonatal death (Klein et al., 1993). As of today, most knowledge of TrkB function comes from primary mouse neuronal cultures and different *Ntrk2*-deficient mice. Although mouse and human TrkB proteins share 90% analogy, relevant models to specifically evaluate the role of human TrkB are scarce if not inexistent, and would provide a necessary tool for identifying and validating the functional efficacy of therapeutics that modulate TrkB activity.

In this study, we used ReNcell^®^ VM, an immortalized human neural progenitor cell line with the ability to differentiate into neurons and glial cells. We first confirmed the expression and functionality of TrkB signaling in these cells. Next, the *NTRK2* gene was disrupted by CRISPR/Cas9 and *NTRK2*-deficient ReNcell VM cells were characterized following neural differentiation. Global RNAseq highlighted a dramatic impact of genetic deletion of *NTRK2* on the expression of specific genes involved in neurogenesis and gliogenesis. *NTRK2*^–/–^ ReNcell VM offer a unique and readily available opportunity to explore the consequences of TrkB loss of function in a controlled laboratory environment to unravel the molecular interactions and signaling pathways involving TrkB.

## 2 Materials and methods

### 2.1 Cell culture

The ReNcell VM cell line was purchased at Merck (#SCC008). The ReNcell VM cell line was maintained in Neurobasal medium (Thermo Fisher #21103-049) supplemented with B-27 (Thermo Fisher #17504-044), MEM Non-essential amino-acids (Thermo Fisher #11140-035), 1% Penicillin Streptomycin (Gibco #15070-063), Glutamax (Thermo Fisher #35050-038), 1 mM Sodium pyruvate (Thermo Fisher #11360-039), 20 ng/ml EGF (Life Technologies #PHG0313) and 20 ng/ml bFGF (R&D #233-FB). Cells were cultivated on Matrigel coated dish. Standard Differentiation (StD) was performed by suppressing EGF and FGF in culture medium.

### 2.2 Quantitative real time polymerase chain reaction (qPCR)

RNA extraction was performed using RNeasy mini kit (Qiagen #74104), with DNase I treatment (Qiagen #79254). RNA concentration was measured using Nanodrop 2000c (Thermo Scientific). The cDNA synthesis was performed using 500 ng total RNA using the Takara Prime Script RT reagent kit (#RR037A). Real-time PCR was performed using the SYBR green assay at the Genomics Platform, National Center of Competence in Research Frontiers in Genetics (Geneva, Switzerland), on a 7900HT SDS system from ABI. The efficiency of each primer was assessed with serial dilutions of cDNA. Primer sequences are reported in Table 1. Relative expression levels were calculated by normalization to the geometric mean of two house-keeping genes β2-microglobulin and GAPDH as described in Vandesompele et al. (2002) and expressed as relative expression values or ratio (EΔΔCt).

**TABLE 1:** Sequences of primers used in the study.

Gene	Sequence
NEUROD1	F: 5′-ATCAGCCCACTCTCGCTGTA-3′
	R: 5′-GCCCCAGGGTTATGAGACTAT-3′
ADGRG6	F: 5′-ACAGAGCAAGGTGGCAGAATGG-3′
	R: 5′-5TTGTCCTCTCCAGCACTCAGGT-3′
PDGFRA	F: 5′-TTGACAACCTCTACACCACACTGA-3′
	R: 5′-TCCGGTACCCACTCTTGATCTTAT-3′
CPS1	F: 5′-TTTAGGGCAATGGCTACAGG-3′
	R: 5′-GTTCTGCAAGAGCTGGGTTC-3′
SLC1A2	F: 5′-TATCATCTCCAGTTTAATCAC-3′
	R: 5′-TTCATTCAACATGGAGATGACC-3′
NTKR2	F: 5′-ACCCGAAACAAACTGACGAGT-3′
	R: 5′-AGCATGTAAATGGATTGCCCA-3′
BDNF	F: 5′- CTACGAGACCAAGTGCAATCC -3′
	R: 5′- AATCGCCAGCCAATTCTCTTT -3′
TUBB3	F: 5′- GGCCAAGGGTCACTACACG -3′
	R: 5′- GCAGTCGCAGTTTTCACACTC -3′
MKI67	F: 5′- AAGCCCTCCAGCTCCTAGTC -3′
	R: 5′- TCCGAAGCACCACTTCTTCT -3′
B2M	F: 5′-TGCTCGCGCTACTCTCTCTTT-3′
	R: 5′-TCTGCTGGATGACGTGAGTAAAC-3′
GAPDH	F: 5′-GCACAAGAGGAAGAGAGAGACC-3′
	R: 5′-AGGGGAGATTCAGTGTGGTG-3′

### 2.3 Construction of miRNA-containing plasmids (mirGE) and lentiviral vectors

The plasmids were constructed using the mirGE system previously described (Rousset et al., 2019). mirGE sequence were synthesized and subcloned using *Xba*I and *Bam*HI restriction sites in pENTR Gateway vector. The most efficient mirGE hairpin template sequences targeting NTRK2 was 5′- TCGTTGATGATTTCTAACCTTT-3′. The final lentivector plasmid was generated by Gateway Recombinational Cloning using an LR Clonase II (Invitrogen, Carlsbad, CA, USA)-mediated recombination of a pENTR plasmid containing the human EFs promoter and a lentivector destination cassette (pCWX-R4dESTR2-PC) containing an additional transcription unit encoding for mCherry marker gene upon human PGK promoter. The lentiviral vector was generated using transient transfection of HEK293T cells with the specific lentivector transfer plasmid, the psPAX2 plasmid encoding gag/pol, and the pCAG-VSVG envelope plasmid. Lentivector titration was performed using transduction of HT-1080 cells followed by flow cytometry quantification of mCherry + cells 5 days after transduction. mCherry positive cells were isolated using Beckman Coulter MoFlow Astrios with help of the Flow Cytometry core facility (CMU, Geneva, Switzerland) and pooled. Silencing of NTRK2 was assessed by qPCR in differentiated ReNcell VM.

### 2.4 CRISPR/Cas9 gene editing of *NTRK2*

Plasmid containing guide RNA sequences and repaired template were purchased from Santa Cruz [TrkB CRISPR/Cas9 KO Plasmid (h): sc-400142 and TrkB HDR Plasmid (h): sc-400142-HDR]. Cells were co-transfected using electroporation Nucleofector 2b (Lonza) with 100 μl of nucleofector buffer and 2 μg of plasmid with X-001 program. The yield of transfection was estimated by green fluorescence protein (GFP) fluorescence. After a week, 1.5 μg/mL of puromycin (InvivoGen, ant-pr) was added to the culture medium for 10 days. At day 10, cells expressing RFP cassette were selected and isolated using Beckman Coulter MoFlow Astrios with help of the Flow Cytometry core facility (CMU, Geneva, Switzerland) and a single cell per well is put in 96-well-plates. Clones are grown for 1–3 weeks. Cells were split for either colony expansion or genomic DNA extraction. Genomic DNA was extracted using Qiagen kit (DNeasy blood and tissue extraction kit, 69504) PCR fragments were amplified using following primers (5′–3′): amplicon 1 Forward 5′-CATTCGCATCTAACAAGGAATCTG-3′ and reverse 5′-AGGCTCCAATCTCGGAAATG-3′ and Amplicon 2: Forward 5′-ACAAGCACCGAGGAGTTAAG-3′ and reverse 5′-CGGTGATGTTCTCAGGATCTAC-3′. The respective amplicons were cloned in a sequencing plasmid using TOPO™ TA Cloning™ Kit (Thermo Fisher # 450030). Identification of positive clones was performed by PCR on genomic DNA using primers Forward 5′-CATTCGCATCTAACAAGGAATCTG-3′ and reverse 5′-AGGCTCCAATCTCGGAAATG-3′. The PCR products contain either the cassette puro/RFP or the exon 9 (Figure 2A).

### 2.5 RNA sequencing (RNAseq)

RNA was isolated from ReNcell VM WT and *NTRK2*^–/–^ cells following 6 days of differentiation using four replicates per conditions. Conditions were the following: no BDNF, BDNF 100 ng/mL 3 and 24 h. The cDNA libraries were constructed from 300 ng of total RNA by the Genomic platform of the University of Geneva using the Illumina TruSeq RNA Sample Preparation Kit according to the manufacturer’s protocol. Libraries were sequenced using paired-end (100, stranded, TruSeqHT stranded mRNA) on Illumina HiSeq4000. The sequencing quality control was done with FastQC v.0.11.5 (FastQC).^1^ The reads were mapped with the STAR v.2.7.0 software to the UCSC human hg38 reference. The average mapping rate was 89.08%. Biological quality control and summarization were done with the PicardTools v.1.141 (Picard Tools).^2^ Reads mapping to each gene feature of UCSC hg38 reference was prepared with HTSeq v0.9.1 (htseq-count) (HTseq).^3^ edgeR v. 3.26.8. Briefly, the counts were normalized according to the library size and filtered. The genes having a count above 1 count per million reads in at least four samples were kept for the analysis. The raw gene number of the set is 26,485. The poorly or not expressed genes were filtered out. The filtered data set consists of 14,878 genes. The differentially expressed genes tests were done with a general linear model with negative binomial distribution considering the time course.

GO term and KEGG metabolic pathways enrichment was performed using homemade scripts for the R software. All annotated pathways for Homo sapiens available on WikiPathways database^4^ were used to generate gene sets, as well as the KEGG metabolic pathways (KEGG)^5^ relative to GRCh38.104. Genes were ranked by their calculated fold-changes (decreasing ranking). A gene set analysis using the GSEA package Version 2.2 (Mootha et al., 2003; Subramanian et al., 2005) from the Broad Institute (MIT, Cambridge, MA) was used to analyze the pattern of differential gene expression between the two groups. Gene set permutations were performed 1,000 times for each analysis. The Normalized Enrichment Score (NES) was calculated for each gene set. GSEA results with a nominal FDR < 0.05 and abs (NES) >1 were considered significant.

### 2.6 Immunoblotting

Proteins were extracted from ReNcell VM using RIPA lysis buffer (50 mM Tris, 1% NP40, 0.1% SDS, 0.15 M NaCl, 0.5% Na deoxycholate) supplemented with protease inhibitor (Roche #04693124001) and phosphatase inhibitor (Sigma#P5726). For phospho-ERK analysis, ReNcell were treated with different concentrations of human BDNF for 5 min (PHC7074, Life Technologies).

Protein concentration was estimated by measuring absorbance at 570 nm (Spectramax, Molecular Devices) following reaction with the BCA Protein Assay Kit Pierce™ (Thermo Fisher # 23227) and quantified by comparison of a standard curve of bovine serum albumin (Sigma # A3912). Fourty μg amount of protein extract was diluted in RIPA buffer, complemented with sample buffer 5X (Tris-base pH 6.8 312 mM, Glycerol 50%, SDS 10%, 2-mercaptoéthanol 25%, bromophenol blue 0.01%), heated 5 min at 100°C. Samples were separated in Bolt 4 to 12%, Bis-Tris, 1.0 mm, Mini Protein Gel (Thermo Fisher # NW04125BOX), run at 1 h 100V in Bolt MOPS SDS Running Buffer (Thermo Fisher # B0001), transfer on PVDF 0.45microM Immobilon P membrane (Millipore # IPVH00010) in transfer buffer (Tris-base 25 mM, Glycine 192 mM, methanol 10%, SDS 0.005%) 90 min at 80 V. Membranes were colored with Fast green 0.01% (in methanol 20% and acetic acid 5%). Membranes were blocked in TBST buffer (150 mM NaCl, 50 mM Tris–HCl, pH 7.6, Tween 0.05%) with BSA (Sigma # 3912) 5%. Membranes were incubated with primary antibodies P-ERK Phospho-p44/42 MAPK (Erk1) (Tyr204)/(Erk2) (Tyr187) (D1H6G) Mouse mAb (Cell Signaling #5726), ERK p44/42 MAPK (Erk1/2) (137F5) Rabbit mAb (Cell Signaling #4695) and recombinant monoclonal mouse antibody anti-TrkB AG424 (Roussel-Gervais et al., 2020) and in secondary antibodies Goat Anti-Mouse IgG (H + L)-HRP Conjugate (Bio-Rad #1706516) and Goat Anti-Rabbit IgG (H + L)-HRP Conjugate (Bio-Rad #1706515). Peroxidase-dependent bioluminescence was revealed with ECL Western Blotting (Witec # K-12042-D10), using Fusion Solo (Witec AG).

### 2.7 Real time cell metabolic analysis (seahorse XFe96 analyzer)

The mitochondrial activity of differentiated WT and *NTRK2*^–/–^ ReNcell VM was measured using Seahorse XF Cell Mito Stress Test (Agilent). For this, 30 000 cells were plated into Seahorse XF Cell Culture Plates and differentiated for 5 days. The XFe96 cartridge was hydrated at 37°C overnight. On the following day, water was replaced by pre-heated Seahorse XF Calibrant for 90 min. After removal of the differentiated medium, 180 μl Agilent Seahorse XF DMEM Medium pH 7.4 (# 103575–100) was added to each well and kept for 1 h at 37°C. Oxygen consumption was measured. After 17 min, oligomycin (3 μM final) was added to block ATP-synthase, leading to decreased to mitochondrial oxygen consumption. After another 17 min, the uncoupling compound carbonyl cyanide-4 (trifluoromethoxy) phenylhydrazone (FCCP) (1 μM final) was added to the cells leading to maximal oxygen consumption. Finally, mitochondria activity was poisoned by addition of a mix of rotenone and antimycin A (0.5 μM final) to measure non-mitochondrial oxygen consumption.

### 2.8 Immunofluorescence

Cells were fixed with PBS (Thermo Fisher #14190)/formaldehyde (Sigma #47608) 2%, permeabilized with PBS/Triton (Sigma # 93420) 0.5%, blocked with PBS/fetal calf serum (Thermo Fisher #10270-106). Primary antibodies used were GFAP (mouse, Millipore MAB360) 1/1000; Beta3 Tubulin (rabbit, Biolegend #802001) 1/2000, BLBP (rabbit, Chemicon) 1/1000; Tau (rabbit, Pierce #MN1020) 1/1000, and PDGFRα (Santa Cruz Biotechnology, sc-338) 1/100, Secondary antibodies are donkey anti mouse-488 (Thermo Fisher #A21202) and donkey anti rabbit-555 (Thermo Fisher #A31572), all 1/1000. Cells are also stained with DAPI (Applichem #A4099) 300 nM. Slides werere mounted with Fluorsave (Calbiochem #345789). Microscope used was Axioskop2 (Zeiss), camera is AxioCam HRc (Zeiss).

### 2.9 Protein tyrosine kinase assay

The protein tyrosine kinase activity in lysates from ReNcell VM was quantified using the Pamstation-12^®^ (Pamgene) apparatus. The principle of the assay is to determine the phosphorylation of the substrate peptide immobilized on a Pamchip array by detection of fluorescence following binding to a fluorescein isothiocyanate (FITC)- conjugated PY20 anti-phosphotyrosine antibody. Samples were prepared according to the PTK assay with cells lysates on Pamstation^®^ 12 User Manual. Briefly, cells were homogenized using a cocktail containing phosphatase et protease inhibitors. Five μg of supernatant were incubated with a master mix containing ATP and the fluorescently labeled antibody, and then loaded on the chips presoaked with BSA 2%. The analysis was run for 94 cycles, while the CDD camera recorded images since cycles 32 every 10, 20, 50 100, and 200 ms. Fluorescent data were exported with Pamgene Bionavigator tools and analyzed with an Excel macro developed at the READS facility (Faculty of Medicine, Geneva University). Slope (based on the 5-time point measurement) was calculated for each sample. A peptide was considered phosphorylated when the slope was greater than 0.4.

## 3 Results

**FIGURE 1:**
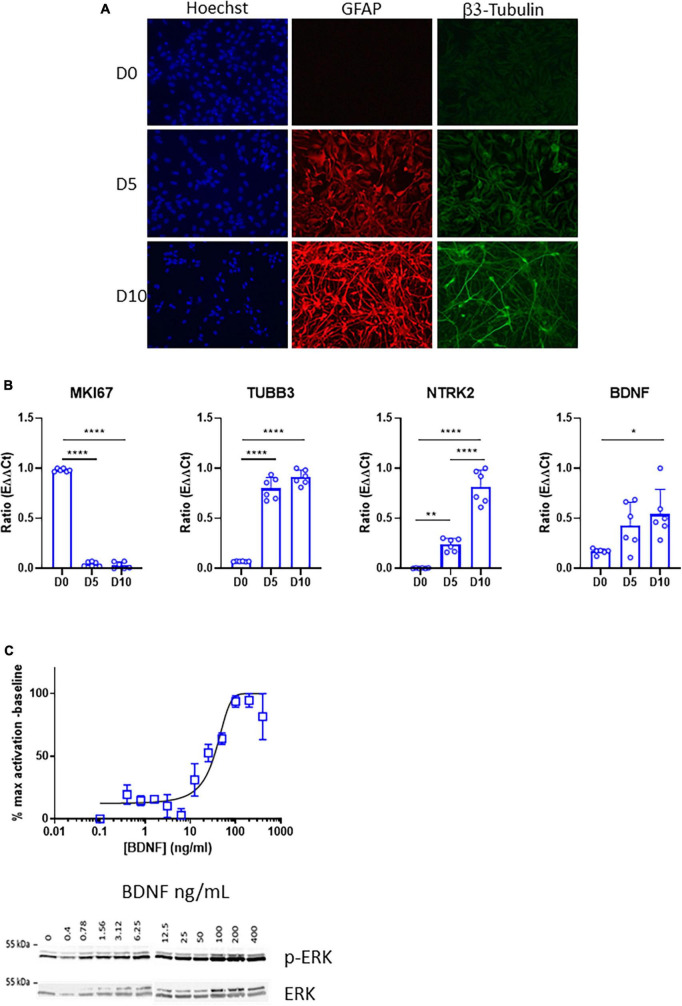
Expression and activity of the TrkB receptor in differentiated ReNcell VM cells. **(A)** Representative images of immunofluorescent staining of ReNcell VM after 5 and 10 days of differentiation. GFAP (red), β3-Tubulin (green), nuclei, Hoechst (blue). Scale bar 200 μm. **(B)** Decrease in RNA levels of the proliferation marker MKI67 during ReNcell VM differentiation is concomitant with upregulation of *TUBB3*, coding for β3-Tubulin, *NTRK2*, the gene coding for TrkB, and *BDNF* in ReNcell VM during differentiation (days 0, 5, and 10). Data represent the mean of six independent experiments performed in triplicates ± SD. Ordinary one-way ANOVA, *p < 0.05, **p < 0.01, ****p < 0.0001. **(C)** Phosphorylation of ERK is induced by treatment with BDNF (5 min) in a concentration-dependent way. Each point was normalized to total ERK and represents the mean of 2–8 independent experiments ± SEM.

ReNcell VM is a human neural stem cell line derived from the ventral mesencephalon of a fetal brain, which was immortalized using the v-myc oncogene (Donato et al., 2007). ReNcell VM was selected for this study because it showed high levels of *NTRK2* expression compared to other commonly used human neural stem cell lines (data not shown) and represents a straightforward system for the study of neurogenesis. In order to characterize TrkB expression during neural differentiation, we used a differentiation protocol allowing generation of neurons, astrocytes and oligodendrocytes upon removal of proliferation growth factors EGF and bFGF. Efficient neuronal and glial differentiation was verified at days 5 and 10 by the presence of high immunofluorescent signals for specific markers such as β3 tubulin (neurons) and Glial Fibrillary Acidic Protein (GFAP) (astrocytes) (Figure 1A). Differentiation efficiency was also confirmed by qPCR analysis showing increased expression of the neuronal marker *TUBB3*, the gene coding for β3 tubulin while the proliferation marker *MIK67* was drastically down-regulated. Importantly, *NTRK2*, the gene coding for TrkB, and *BDNF*, the main TrkB ligand, were upregulated at day 5 and further elevated at day 10 (Figure 1B). Phosphorylation of extracellular signal-related kinase (ERK) is a key downstream target of TrkB activation (Revest et al., 2014). Accordingly, phospho-ERK/total ERK ratio increased in a dose-dependent way in differentiated ReNcell VM (10 days) 5 min after BDNF addition (Figure 1C). Altogether, these data revealed that a functional TrkB/BDNF signaling was present following neural differentiation of ReNcell VM.

**FIGURE 2:**
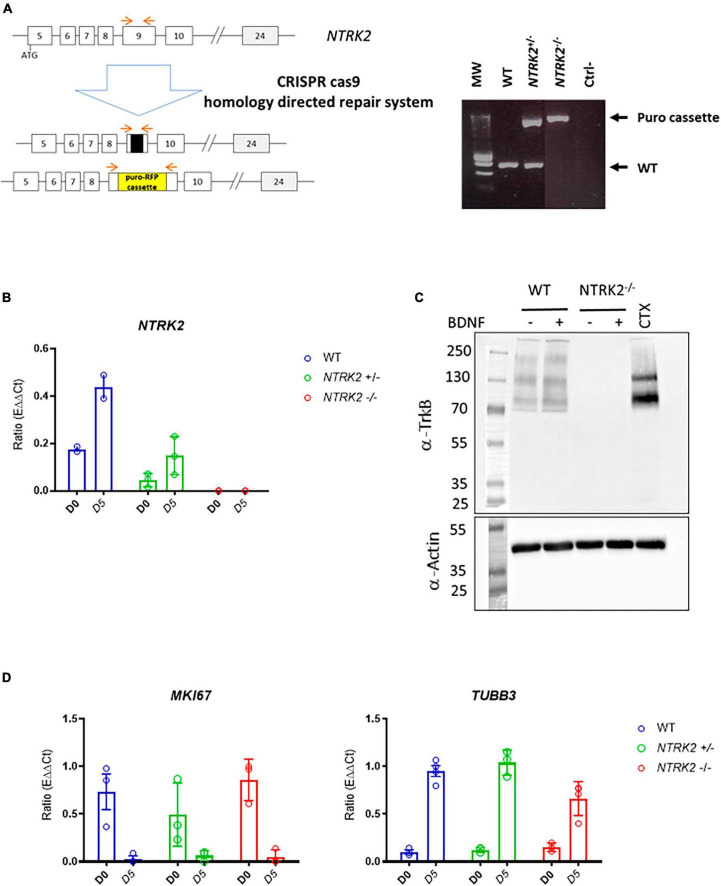
Generation and validation of *NTRK2*^–/–^ ReNcell VM cells. **(A)** Schematic representation of the CRISPR/Cas9 strategy leading to insertion of a puromycin resistance gene in exon 9 of the *NTRK2* gene and selection by PCR. Red arrows indicate localization of amplification primers leading to detection of WT (lower band), heterozygous *NTRK2*^±^ (2 bands) and homozygous *NTRK2*^–/–^ clones (higher band). Ctrl- = H_2_O control **(B)**. NTRK2 RNA was absent in *NTRK2*^–/–^ cells and reduced in *NTRK2*^±^ cells compared to WT. Data represents the mean of three independent experiments performed in triplicates ± SD. **(C)** Immunoblot using an anti-TrkB monoclonal antibody showing detection of three bands in ReNcell VM, which are absent in *NTRK2*^–/–^ cells; human cortex was used as positive control. Lower part of the gel shows the immunoblotting of β actin, used as a loading control **(D)**. Similar pattern of RNA levels of the proliferation marker MKI67 and β3-Tubulin in WT, *NTRK2*^±^, and *NTRK2*^–/–^ clones during differentiation (days 0 and 5). Each point represents the mean of four independent experiments ± SEM.

**FIGURE 3:**
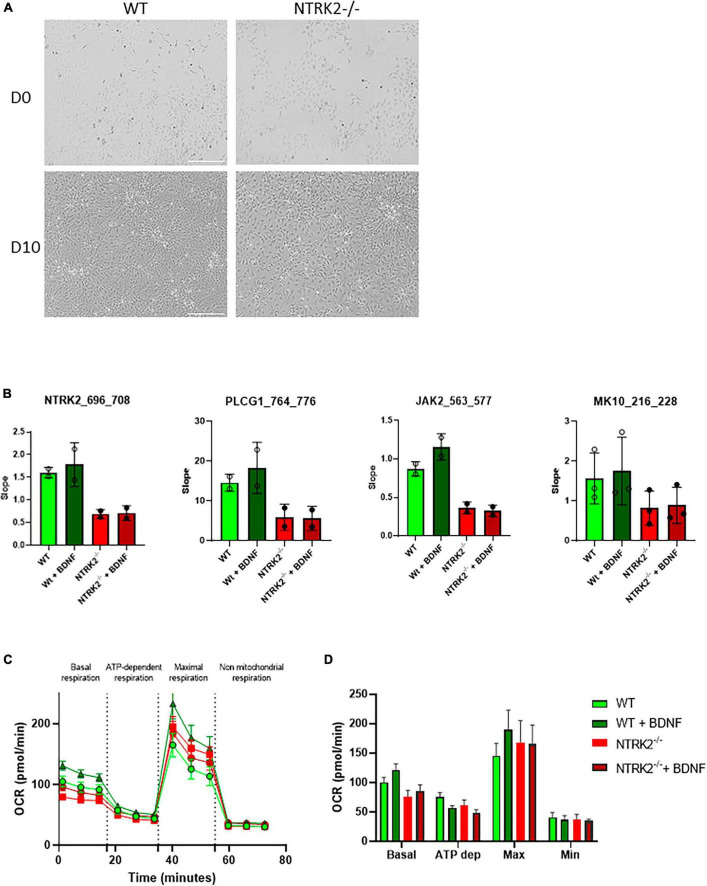
Functional characterization of *NTRK2*^–/–^ ReNcell VM cells. **(A)** Representative image of phase contrast microscopy of WT and *NTRK2*^–/–^ ReNcell VM. Scale bar 200 μm. **(B)** Functional kinase assay showing decreased phosphorylation of selected peptides (aa numbering shown in brackets) by protein kinases active in cell lysates in *NTRK2*^–/–^ ReNcell VM. Data are shown as the mean of 2–3 independents experiments performed in triplicates ± SD. **(C)** Kinetics course of OCR as seen in the Seahorse XF Cell Mito Stress assay in differentiated WT and *NTRK2*^–/–^ ReNcell VM. **(D)** Bar graphs representing the average of three time points for each measured phase during Seahorse XF Cell Mito Stress assay in WT and *NTRK2*^–/–^ ReNcell VM; Data of **(C,D)** represent the mean of four independents experiments performed in at least six replicates ± SEM. Ordinary one-way ANOVA did not detect statistical differences between groups.

The *NTRK2* gene encodes several splice variants leading to full-length TrkB (TrkB-FL) and three truncated forms (TrkB-T1, TrkB-T2, and TrkB-T-ShC), which lack an intracellular tyrosine kinase-signaling domain (Li et al., 2023). In order to generate a full *NTRK2* knockout in ReNcell VM, we used the CRISPR/Cas9 technology with TrkB CRISPR/Cas9 KO and HDR plasmids to insert a puromycin resistance gene (Puro) and a Red Fluorescent Protein (RFP) gene in *NTRK2* exon 9, common to all the above-mentioned TrkB isoforms. Upon clonal selection, we identified that most clones displayed heterozygous deletion (*NTRK2*^±^) while 2 clones were identified to contain a Puro cassette inserted on both *NTRK2* alleles (*NTRK2*^–/–^) (Figure 2A). qPCR experiments showed that NTRK2 expression was absent in *NTRK2*^–/–^ cells while reduced NTRK2 expression was observed in *NTRK2*^±^ compared to WT, suggesting that the insertion of the Puro cassette led to instability of the NTRK2 transcripts (Figure 2B). The impact of Puro insertion on TrkB protein expression was validated by Western blot using a monoclonal antibody directed to the extracellular region of TrkB (Subramanian et al., 2005). The monoclonal antibody detected 3 bands in ReNcell VM and 2 in human cortex. The lower band most likely corresponds to isoforms TrkB.T1 (90 kDa), the medium band to TrkB-FL (140 kDa), while the upper band (around 200 kDa) may correspond to a dimer (Luberg et al., 2010). All bands were absent in differentiated *NTRK2*^–/–^ cells, confirming deletion of TrkB in *NTRK2*^–/–^ cells (Figure 2C). Interestingly, the differentiation process of ReNcell VM appeared mildly affected by TrkB loss of function as qPCR experiments indicated that the proliferation marker *MIK67* was down-regulated in both WT and *NTRK2*^–/–^ cells while neuronal *TUBB3* was upregulated upon differentiation in both cell lines, although slightly lower in *NTRK2*^–/–^ cells (Figure 2D). *NTRK2*^–/–^ cells had a similar observable morphology compared to WT cells (Figure 3A). However, phospho-ERK was drastically diminished in *NTRK2*^–/–^ cells at basal state and even following BDNF treatment, highlighting the importance of TrkB-mediated ERK phosphorylation in ReNcell VM (Figure 3B) and highlighting the functional loss-of-function of TrkB. Upon activation, TrkB displays a phosphotyrosine kinase activity, which phosphorylates its intracellular region (autophosphorylation) and other cytosolic targets. In order to address the impact of *NTRK2*-deficiency on kinase activity present in cell homogenates of WT and *NTRK2*^–/–^ ReNcell VM, we performed kinase activity profiling using a PamChip^®^ peptide arrays. Among known downstream targets of TrkB activation, the following selected peptides were phosphorylated: (i) a TrkB peptide containing Tyr706 and 707 (Fred et al., 2019); (ii) a phosphoinositide phospholipase C (PLCG1) peptide containing Tyr771 (Sekiya et al., 2004) and 775 (Serrano et al., 2005); (iii) a Janus kinase 2 (JAK2) Tyr570 peptide (Lin et al., 2006) and a Mitogen-activated protein kinase 10 (MK10) (aka c-Jun N terminal kinase) Tyr223 and Tyr228 peptide (Escudero et al., 2019; Figure 3B). All selected peptides displayed the same pattern of phosphorylation as the one observed for ERK by immunoblotting. Extracts from WT cells showed much higher phosphorylation than *NTRK2*-deficient cell extracts, and a small phosphorylation increase upon addition of BDNF, which was not present in *NTRK2*^–/–^ cell extracts. Altogether, this suggests that tyrosine kinase activity present in WT is highly dependent on TrkB and that basal BDNF or other TrkB activating factors are secreted by differentiated ReNcell VM inducing almost maximal levels of TrkB activation.

Next, the functional consequences of TrkB loss-of-function on neuronal metabolism were explored. Neural cells are highly dependent on mitochondria for energy production through aerobic oxidative phosphorylation (Rangaraju et al., 2019). Mitochondria are very efficient organelles in utilizing oxygen (O_2_) and substrates, mostly derived from glucose, to generate cellular energy in the form of adenosine triphosphate (ATP) (Mishchenko et al., 2018), and the BDNF/TrkB axis contributes to metabolic homeostasis (Nakagomi et al., 2015). In order to evaluate the impact of *NTRK2* deletion on cellular metabolism, we performed a Seahorse XF Cell Mito Stress Test, which measured the oxygen consumption rate (OCR) in live differentiated (5 days) WT and *NTRK2*^–/–^ cells following treatment with different pharmacological agents (oligomycin, FCCP and a mix of rotenone and antimycin A) (Figure 3C). Basal mitochondrial respiration showed a slight, although not significant, increase of OCR following BDNF treatment in WT cells, which was absent in *NTRK2*^–/–^ cells (Figure 3D). Injection of oligomycin decreased OCR to similar level in all investigated groups. FCCP injection led to a significant increase in OCR in all groups with a slight increase in WT BDNF treated cells, which was absent in *NTRK2*^–/–^ cells. Rotenone and antimycin A mixture decreased OCR in all tested groups to similar level (Figure 3D). These data indicate that deletion of TrkB did not affect the mitochondrial metabolism of ReNcell VM and confirmed that BDNF leads to a slight increase of mitochondrial function.

**FIGURE 4:**
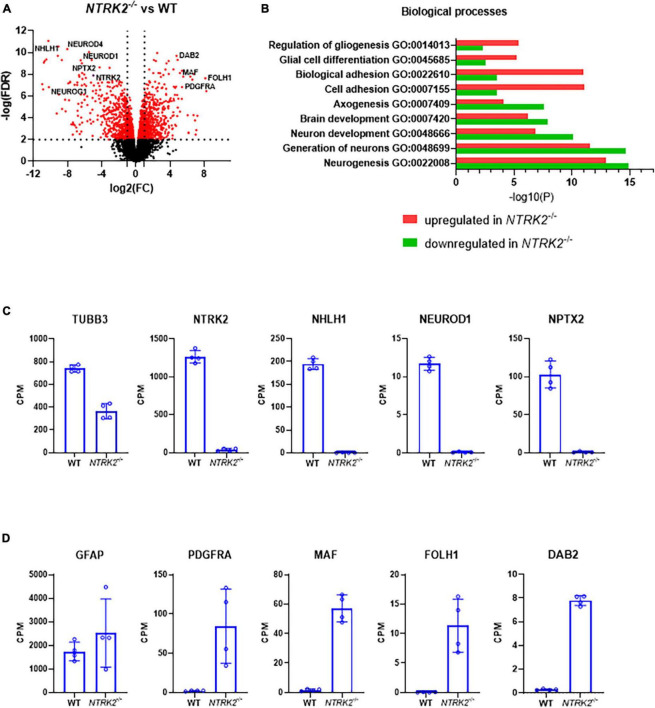
*NTRK2* deficiency leads to transcriptomic changes of specific neurogenesis and gliogenesis pathways in differentiated ReNcell VM. **(A)** Volcano plot comparing WT and *NTRK2*^–/–^ ReNcell VM. Several statistically significant representative genes involved in neurogenesis and gliogenesis are highlighted. **(B)** Graph representing the most significantly modified biological pathways in WT and *NTRK2*^–/–^ ReNcell VM. Selected gene expression data of the RNAseq highlighted massive transcriptomic changes in genes regulating neuronal differentiation **(C)** and early glial markers **(D)**; *n* = 4 ± SD. -logFDR < 2, expect ns (not significant); CPM: counts per million.

In order to address the impact of *NTRK2* deletion on ReNcell VM differentiation, we performed bulk RNAseq to compare WT and *NTRK2*^–/–^ ReNcell VM after 6 days of differentiation. Altogether, 14,275 genes were identified with 594 down- and 633 up-regulated genes (FDR < 2) (Figure 4A). Interestingly, addition of BDNF (100 ng/mL) for 24 h did not induce significant gene expression [available at GEO Submission (GSE242199)], suggesting either that sufficient BDNF was generated in the system to saturate the signaling pathway or that BDNF is not a major inducer of transcription changes–as previously seen in other studies (Alder et al., 2003). The most differentially expressed genes in *NTRK2*^–/–^ cells were documented in Tables 2, 3. In these tables, single cell RNAseq data from the human protein atlas^6^ were used to determine brain specificity and cell type enrichment in the adult brain. Most upregulated genes in *NTRK2*^–/–^ cells were expressed in oligodendrocyte progenitor cells, oligodendrocytes and astrocytes while downregulated genes mostly showed neuronal expression. However, specific markers of oligodendrocytes including *SOX10*, *NKX2*, *OLIG1*, and *OLIG2* were not detected.

**TABLE 2:** Ten most upregulated protein coding genes in *NTRK2*^–/–^ ReNcell VM.

Gene	Log2FC	Adjusted *p*-value	Brain specificity	Enriched brain cell expression (adult)
FOLH1	8.93	1.16087E-05	Yes (+ prostate)	Oligo
CDH20	6.65	1.03219E-05	Yes	Oligo, OPC, astrocytes
A2M	6.10	0.001974902	No (adipocytes, fibroblasts)	Microglia
PDGFRA	5.61	0.000110783	Yes (+ reproductive system)	OPC
ZNF503	5.58	1.48106E-07	No (epithelial cells)	Neurons, OPC, microglia
DOK6	5.53	7.25049E-07	Yes	Neurons, OPC
KCNK2	5.08	0.000104561	Yes	Neurons, OPC, microglia
SOD3	4.98	8.53231E-06	No (fibroblasts, muscle)	Astrocytes, microglia
MAF	4.97	2.2123E-07	No (immune cells and others)	Neurons
SFRP2	4.84	5.75258E-06	No (fibroblasts)	Astrocytes, neurons

Oligo, oligodendrocytes; OPC, oligodendrocyte progenitor cells.

**TABLE 3:** Ten most downregulated protein coding genes in *NTRK2*^–/–^ ReNcell VM.

Gene	Log2FC	Adjusted *p*-value	Brain specificity	Enriched brain cell expression (adult)
MAGEH1	−10.84	1.60804E-08	No	Neurons, oligo, OPC
MGMT	−10.68	4.15432E-06	No (hepatocytes)	Neurons, oligo, OPC, astrocytes
EBF2	−10.43	1.48106E-07	No	Microglia
CMBL	−10.02	4.03068E-06	No (enterocytes)	Neurons
LHX5	−9.25	0.000236301	No	None
NEUROD4	−9.04	3.0599E-07	Yes	Neurons
NRN1	−9.03	1.48106E-07	No (adipocytes)	Neurons
NLRP2	−8.64	7.25049E-07	No	Neurons
NHLH2	−8.63	1.07925E-06	No (skeletal muscle)	Neurons, oligo, OPC
MGST2	−8.00	2.56506E-05	No (liver)	Neurons, microglia

Oligo, oligodendrocytes; OPC, oligodendrocyte progenitor cells.

Pathway analysis indicated important increase in genes involved in gliogenesis and cell adhesion in *NTRK2*^–/–^ ReNcell VM, while genes involved in neuronal differentiation and neurogenesis were prominent in WT cells (Figure 4B). Gene expression data from selected genes were extracted from the RNAseq dataset to illustrate these pathways. NTRK2 expression was absent in *NTRK2*^–/–^ cells, confirming our qPCR data, while key transcription factors involved in neurogenic differentiation *NHLH1*, *NEUROD1*, and *NEUROD2* were only present in WT cells while the maturation marker *TUBB3* was significantly downregulated in *NTRK2*^–/–^ cells (Figure 4C). On the other hand, specific markers of glial progenitors *PDGFRA*, *MAF*, *FOLH1*, and *DAB2* were enriched in *NTRK2*^–/–^ cells (Figure 4D). Interestingly, the typical astrocyte marker *GFAP* was not significantly dysregulated. As the RNAseq was performed on a single NTRK2^–/–^ clone, we aimed at excluding that our observation may have derived from a clonal effect of the selected clone. We used an alternative approach to address the transcriptional impact of mitigation of TrkB by expressing a microRNA specifically targeting *NTRK2* in ReNcell VM using the mirGE technology (Rousset et al., 2019). The microRNA targeting *NTRK2* showed high efficiency as *NTRK2* mRNA was decreased by 90%. These cells showed significant down-regulation of the neuronal genes *NeuroD1* and *NPTX2* and up-regulation of the glial marker *SLC1a* similar to NTRK2^–/–^ cells (Supplementary Figure 1). These data support that the observed impact on neuronal genes and early glial gene expression is due to TrkB loss of function and not to a pleiotropic clonal effect of the TrkB knockout ReNcell.

**FIGURE 5:**
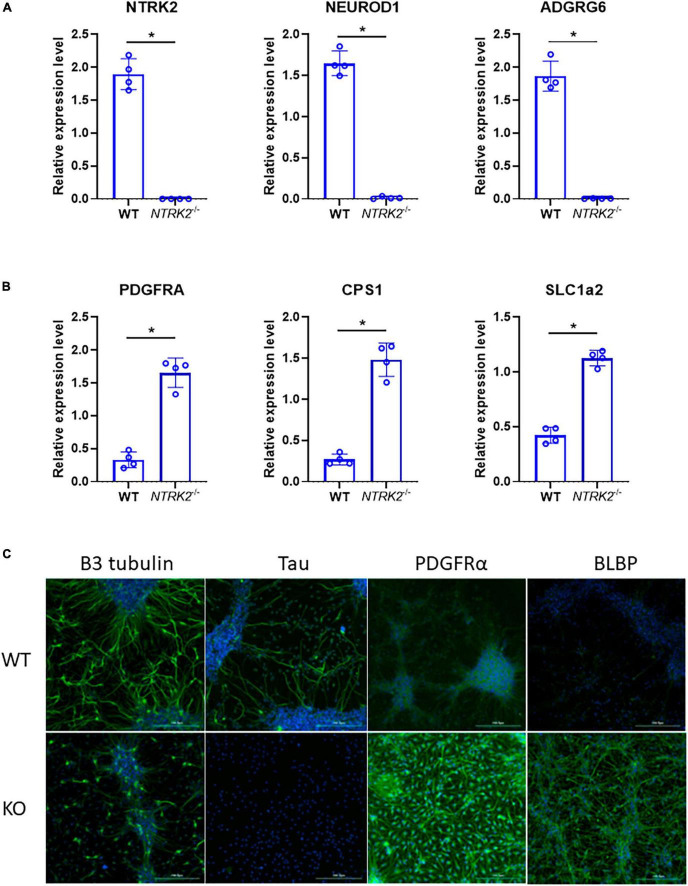
Validation of RNAseq data by qPCR and immunofluorescence. **(A,B)** Gene expression of selected genes by qPCR. Data represent the mean of four independent experiments performed in triplicates ± SD. Two-tailed Mann-Whitney non-parametric test, *p < 0.05. **(C)** Immunofluorescence analysis of differentiated WT and *NTRK2*^–/–^ cells highlighting enrichment of early glial markers PDGFRα and BLBP in *NTRK2*^–/–^ cells and decreased neurogenesis as observed by absence of Tau and decreased β3 tubulin. Various markers are shown in green, blue- DAPI. Scale bar 200 μm.

Finally, as confirmatory experiment, another batch of differentiated ReNcell VM was tested by qPCR using selected neuronal genes (*NTRK2*, *NEUROD1*, and *ADGRG6*) and glial genes (*PDGFRA*, *CPS1*, and *SLC1A2*), which were identified in the RNAseq dataset (Figures 5A, B). In addition, immunofluorescent staining was performed using differentiated WT and *NTRK2*^–/–^ cells (Figure 5C). The immunofluorescent signal for neuronal marker β3-tubulin and Tau labeled long neuronal processes, which were absent in *NTRK2*^–/–^ cells. Antibodies directed against the early glial markers brain lipid-binding protein (BLBP) and the oligodendrocyte precursor cell marker PDGFRα labeled almost exclusively cells from *NTRK2*^–/–^ ReNcell VM. Overall, changes observed in immunostaining were consistent with RNAseq data.

Altogether, our data show that ReNcell VM is a relevant neural cell line to study the BDNF/TrkB axis. *NTRK2* deletion did not impact metabolic activity of differentiated ReNcell VM, but impaired proper neuronal differentiation leading to increased presence of glial and cell adhesion markers. This suggests that *NTRK2*^–/–^ ReNcell VM did not develop normally as it was enriched with progenitor glial cells, the first glial cell population appearing during brain ontogeny, which serve as scaffold for neuronal migration in the developing brain.

## 4 Discussion

In this study, we characterized the function and expression of TrkB in ReNcell VM and generated a *NTRK2*-deficient ReNcell VM to study the role of TrkB. We confirmed that ReNcell VM progenitors differentiated efficiently into a mixed culture of neurons and glial cells leading to high expression of NTRK2 RNA already after 5 days of neural differentiation, confirming a previous proteomic study (Song et al., 2019). We synthesized a recombinant monoclonal antibody AG424 previously developed as a specific TrkB agonist (Wang, 2008). This monoclonal antibody was specific for TrkB as it detected 3 bands of equal density in differentiated ReNcell VM, which were absent in *NTRK2*^–/–^ cells. The 3 bands likely represent the full length TrkB, the kinase deficient TrkB-T1 and possibly a heterodimer of the above. The TrkB-T1 isoform is mainly expressed in astrocytes and is the most abundant TrkB receptor in the adult brain. Although its exact function is still cryptic (Tessarollo and Yanpallewar, 2022), TrkB-T1 is often described as a dominant-negative inhibitor of the full-length TrkB, as it does not transduce the signal through the intracellular kinase motif following ligand activation (Tomassoni-Ardori et al., 2019). In spite of substantial TrkB-T1 expression, we highlighted a significant tyrosine kinase activity of ERK and several downstream targets of TrkB, which were strikingly down-regulated in *NTRK2*-deficient cells. Nevertheless, in WT cells, it is possible that truncated TrkB-T1 isoform may substantially inhibits TrkB, as the addition of BDNF did not result in significant transcriptomic or metabolic changes. However, it is increasingly recognized that TrkB-T1 is not solely a negative competitive regulator of TrkB, as BDNF-dependent activation of TrkB-T1 leads to the release of calcium from intracellular stores and the activation of specific downstream events (Rose et al., 2003), including important changes in gene expression (Pattwell et al., 2020). It is also possible that the concentration of BDNF (100 ng/mL) used in this study was too low or that BDNF-induced changes were transient (Alder et al., 2003) and therefore not observable at the 24-h end-point. However, no significant transcriptomic changes were detected after 3 h of BDNF treatment (data not shown). In addition, as ReNcell VM express BDNF, the endogenous BDNF may be sufficient for TrkB activation, leading to its internalization thereby limiting its activity (Du et al., 2003). The recombinant human TrkB-Fc chimera protein, which potently blocks BDNF activity (Fulgenzi et al., 2015, 2020; Nair et al., 2017; Montroull et al., 2019) may help resolving the impact of endogenous BDNF in the TrkB-mediated signaling of ReNcell VM.

This study is the first, to our knowledge, to generate an effective, complete and validated knock-out of TrkB in human cells. The CRISPR/Cas9 approach used in this study disrupted all TrkB isoforms, but no obvious impact on ReNcell VM morphology, survival and metabolic function was observed. However, following differentiation, important transcriptomic changes were observed in *NTRK2*-deficient cells, including a massive decrease in specific genes associated with neuronal development, as expected from the strong neurodevelopmental defects observed in Ntrk2 knockout mice (Klein et al., 1993). For example, RNA levels of key early transcription factors involved in neurodevelopment such as *NEUROD1*, *NEUROD4*, and *NEUROG2* were virtually absent in differentiated *NTRK2*^–/–^ ReNcell VM. On the other hand, this is, to our knowledge, the first description of an inhibitory role of TrkB on the expression of genes associated with gliogenesis and cell adhesion. Indeed, several genes enriched in oligodendrocyte neural precursors and involved in neuronal migration (*FOLH1*, *DAB2*, *MAF*) were only expressed in *NTRK2*^–/–^ ReNcell VM. Among well-known markers of gliogenesis, *PDGFRA* is a receptor tyrosine kinase often used to identify oligodendrocyte precursors during development and a well-described regulator of oligodendrocytes and astrocyte development (Montroull et al., 2019). Interestingly, the typical *GFAP* marker was only slightly upregulated in *NTRK2*^–/–^ ReNcell VM, suggesting that different glial differentiation pathways were affected.

A handful of studies analyzed the transcriptomic changes induced by BDNF in neurons. BDNF treatment of primary mouse hippocampal neurons revealed rapid increase in the expression of several immediate early genes, such as Fos or Egr1, while Vgf (VGF Nerve Growth Factor Inducible) showed more sustained upregulation (Alder et al., 2003). Further studies confirmed that the above-mentioned genes are indeed upregulated in BDNF-treated neurons derived from human induced pluripotent stem cells (Traub et al., 2017; Merkouris et al., 2018; Liu et al., 2023). To our knowledge, only one study analyzed transcriptomic changes in Ntrk2-deficient cells. Maynard et al used mice with selective deletion of TrkB in cortistatin interneurons (Maynard et al., 2020). They performed an analysis of bulk RNAseq in cortices of 21-days-old mice, which corresponds to the time when they develop spontaneous epileptic seizures (Hill et al., 2019). Important gene dysregulation was observed, including significant elevation of secretogranin II (Scg2) and neuronal pentraxin II (Nptx2) and decreased Gfap in *Ntrk2*-deficient neurons. While one would expect NTRK2^–/–^ cells to show a decrease in BDNF-induced genes, our RNAseq dataset did not identify similar pathways or changes in the genes mentioned above (data not shown) even for *VGF* or *SCGF*. This variation with previous studies highlights experimental differences, such as the impact of an epileptic seizure in the cortex of Ntrk2-deficient interneurons. A key limitation of our approach was the use of bulk RNAseq of an heterogenous cellular system containing glial cells possibly enriched in TrkB.T1 and neuronal cells possibly enriched in full-length TrkB. Global RNA changes therefore represent the average of the combined effects of theses isoforms. Further, the striking differences between WT and *NTRK2*^–/–^ ReNcell VM may be amplified by an early differentiation leading to a switch toward glial differentiation in *NTRK2*^–/–^ cells and neurons in WT cells. Single cell RNAseq at different stages of differentiation may help resolve this issue.

Overall, we have confirmed a key role of TrkB in neural differentiation, highlighting the importance of this receptor in brain development and function and supporting therapeutic potential of strategies targeting the BDNF/TrkB system for neuroprotection, cognitive enhancement and mitigation of neurodegenerative diseases (Azman and Zakaria, 2022). With the recent discovery that antidepressants and psychedelics directly bind to TrkB dimers to facilitate BDNF action and neuronal plasticity, the rationale for developing drugs targeting TrkB gained much interest (Casarotto et al., 2021; Moliner et al., 2023). The *NTRK2*^–/–^ ReNcell represent an optimal tool for testing the specificity of novel small molecules agonists or selective TrkB positive allosteric modulators. Future studies using this cell line will allow to address the impact of TrkB deletion on the proportion of neuronal and glial markers as well as to further evaluate the effect of TrkB signaling on neuronal function under controlled conditions, such as ReNcell derived organoids, and in homeostatic conditions or the presence of disease triggers.

## Data availability statement

The datasets presented in this study can be found in online repositories. The names of the repository/repositories and accession number(s) can be found below: https://www.ncbi.nlm.nih.gov/, GSE242199.

## Ethics statement

Ethical approval was not required for the studies on humans in accordance with the local legislation and institutional requirements because only commercially available established cell lines were used.

## Author contributions

AR-G: Conceptualization, Data curation, Formal analysis, Investigation, Methodology, Writing – original draft, Writing – review and editing. SS: Data curation, Investigation, Methodology, Writing – review and editing. YC: Data curation, Investigation, Methodology, Writing – review and editing. SL: Data curation, Methodology, Software, Writing – review and editing. TS: Conceptualization, Funding acquisition, Writing – review and editing. K-HK: Funding acquisition, Resources, Writing – review and editing. VJ: Funding acquisition, Resources, Conceptualization, Data curation, Formal analysis, Project administration, Validation, Writing – original draft.

## Footnotes

1
http://www.bioinformatics.babraham.ac.uk/projects/fastqc/

2
http://picard.sourceforge.net/

3
https://htseq.readthedocs.io/en/latest/

4
http://www.wikipathways.org/index.php/WikiPathways

5
http://www.genome.jp/kegg/

6
proteinatlas.org

## Supplementary material

Supplementary Figure 1 Genetic down-regulation of NTRK2 by a mirGE microRNA impacts neuronal and glial markers similarly to NTRK2^–/–^ cells. Gene expression of selected genes by qPCR. Data represent the mean of 4 independent experiments performed in triplicates ± SD. Ordinary one-way ANOVA multiple comparison, *****p* < 0.0001.
