# Supplementary figures and images for: Genetic knockout of NTRK2 by CRISPR/Cas9 decreases neurogenesis and favors glial progenitors during differentiation of neural progenitor stem cells
Source: Front Cell Neurosci. 2023 Dec 14;17:1289966. doi: 10.3389/fncel.2023.1289966 (PMC10757602; doi:10.3389/fncel.2023.1289966)

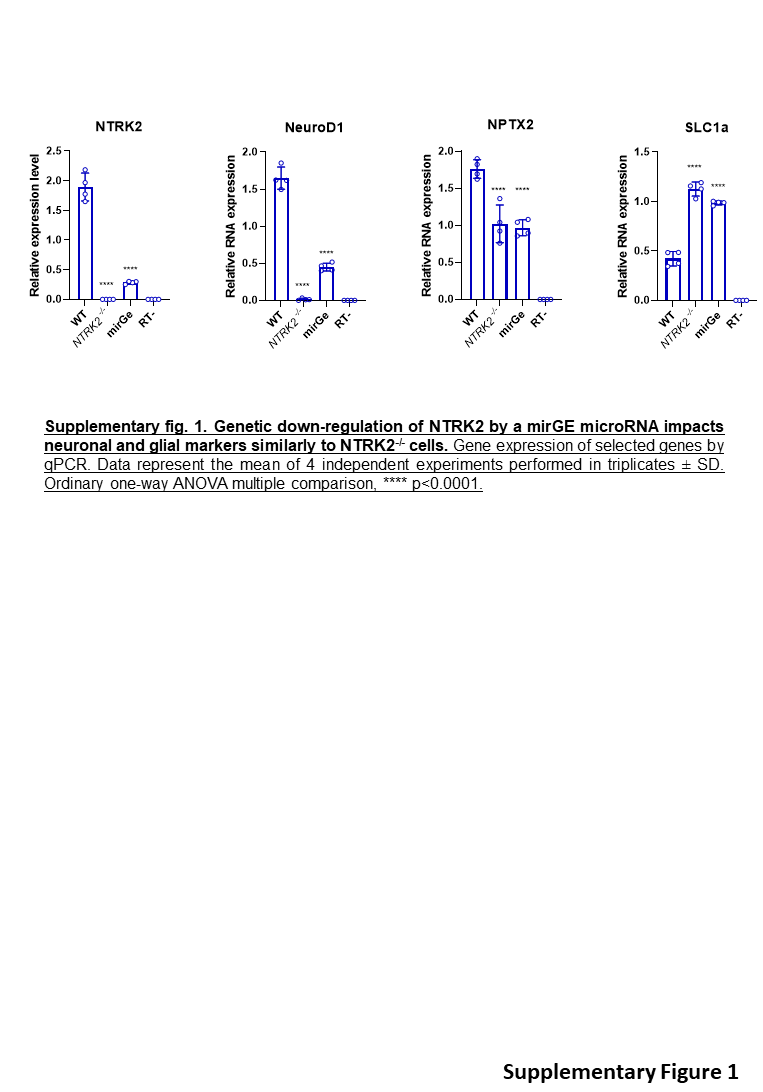

Supplement: Supplementary Figure 1 — Genetic down-regulation of NTRK2 by a mirGE microRNA impacts neuronal and glial markers similarly to NTRK2–/– cells. Gene expression of selected genes by qPCR. Data represent the mean of 4 independent experiments performed in triplicates ± SD. Ordinary one-way ANOVA multiple comparison, ****p < 0.0001. [file Image_1.tif]
